# The Role of RIPK1/3 in Adult Onset Still’s Disease Patients With Liver Damage: A Preliminary Study

**DOI:** 10.3389/fimmu.2020.560744

**Published:** 2020-11-24

**Authors:** Xuesong Liu, Ruru Guo, Xinyu Meng, Jianchen Fang, Liangjing Lu

**Affiliations:** ^1^ Department of Rheumatology, Renji Hospital, School of Medicine, Shanghai Jiaotong University, Shanghai, China; ^2^ Department of Ultrasound, Renji Hospital, School of Medicine, Shanghai Jiaotong University, Shanghai, China; ^3^ Department of Pathology, Renji Hospital, School of Medicine, Shanghai Jiaotong University, Shanghai, China

**Keywords:** adult onset Still’s disease, receptor interacting protein kinase, liver, lymphocyte, adaptive immune

## Abstract

**Objective:**

This study aimed to investigate the distributions of lymphocytes in adult onset Still’s disease (AOSD) with liver dysfunction, and further prospectively explore whether receptor interacting serine/threonine kinases (RIPKs) in lymphocytes play a role in the pathogenesis of AOSD especially liver involvement.

**Methods:**

Seventy-two AOSD patients and 19 cases of healthy controls (HCs) were retrospectively reviewed, the AOSD group was then divided into liver damage (LD) group and non-liver damage (NLD) group, and the distributions of lymphocytes in peripheral blood were analyzed. Another independent 24 AOSD patients and 20 HCs were recruited for prospective study of RIPKs; the RIPKs in peripheral blood lymphocytes were detected by flow cytometry. Liver biopsy specimens were obtained from two AOSD patients and underwent immunochemistry analysis with RIPK1 and RIPK3 antibody.

**Results:**

In the retrospective study, AOSD showed significantly abnormal lymphocytes distributions, and disease activity was positively correlated with percentage of CD3^+^ T cells. LD patients were younger in age and showed higher disease activity score than NLD patients; they had higher frequencies of CD3^+^ T cells, especially higher CD8^+^ T cells (all *p*<0.05). In the prospective study, RIPKs in lymphocytes were significantly higher in AOSD patients than that of HCs, and LD patients also showed higher RIPKs expression than NLD patients. In addition, RIPKs were positively correlated with erythrocyte sedimentation rate (ESR) and disease activity in AOSD patients and LD and NLD subgroups (all *p*<0.05). Further, RIPKs expression was confirmed in two AOSD patients’ liver. ROC curve analysis indicated that RIPKs in lymphocytes (%) could be potential biomarkers in the diagnosis of AOSD and liver damage.

**Conclusions:**

Abnormal lymphocytes distributions and RIPKs expression were detected in AOSD. Aberrant expression of RIPKs in lymphocytes might be involved in the pathogenesis of AOSD. RIPKs could be candidate markers for AOSD and liver damage.

## Background

Adult-onset Still’s disease (AOSD) is a rare systemic inflammatory disorder of unknown etiology, characterized by persistent or intermittent hyperthermia, easy-to-subside rash, arthritis, and varying degrees of multiple organ involvement (1). However, the symptoms and laboratory findings of AOSD are not disease-specific; diagnosis and precise detection of disease activity is difficult (2).

The pathogenesis of AOSD is complex and it is well known that activated innate immunity plays a major role in AOSD pathogenesis (3, 4), in particular, neutrophil and macrophage activation is the hallmark of AOSD (5). However, the role of lymphocytes in AOSD pathogenesis has so far escaped a detailed understanding in AOSD (6). In our clinical work, we noticed there were abnormal distributions of lymphocytes in AOSD patient, which indicated that survival and death of lymphocytes might be involved in the process of the disease.

Receptor interacting serine/threonine kinase1 and 3 (RIPK1and RIPK3) are key regulators of cell death and survival (7–9). Studies showed that RIPK1 and RIPK3 could regulate death ligands (such as TNF, FASL) and then activate Caspase 8 to induce apoptosis of immune cells and relieve inflammatory response (10, 11); Whereas Zhu et al. (12) reported that RIPK1 expression in PBMCs from patients with systemic lupus erythematosus (SLE) was negatively correlated with the disease activity index, which suggested that RIPK1 was involved in the immunopathogenic injury in SLE. Recently published studies revealed that human non-cleavable RIPK1 variants promoted activation of RIPK1, and led to an increased inflammatory response in PBMCs, which resulted in an early-onset periodic fever syndrome and severe intermittent lymphadenopathy (13, 14). Now AOSD is categorized as a multigenic autoinflammatory disorder at the crossroads of autoinflammatory and autoimmune diseases (15). However, the role of receptor interacting serine/threonine kinases (RIPKs) in AOSD has not yet been investigated.

A previous genetic study suggested that RIPKs were implicated in the pathogenesis of liver disease by regulating caspase-dependent hepatocyte apoptosis, and RIPK1/3 participated in many experimental liver disease models (9, 16). Liver involvement in AOSD is quite common, with up to 50–75% of patients presenting with hepatomegaly or elevated liver enzymes (17). So we are wondering if liver damage of AOSD and RIPKs are somehow linked. To the best of our knowledge, no report has described their relationship.

Therefore, in our preliminary study, we aimed to investigate whether lymphocytes distribution sand RIPK1/3 expression in lymphocytes associated with AOSD activity, and to discuss whether RIPKs are involved in the liver damage of AOSD. We also sought to identify whether RIPKs can be potential diagnostic markers for AOSD and liver damage.

## Methods

### Subjects

Seventy-two patients diagnosed as AOSD and 19 cases of healthy control (HC) in our hospital from June 2001 to June 2017 were retrospectively reviewed. AOSD was diagnosed according to Yamaguchi criteria (18). Patients who had infections, hematological, and other autoimmune diseases were excluded from this study. The clinical features of patients and HC were shown in **Table 1**. The 72 patients were further divided into liver damage (LD) group (n=29) and non-liver damage (NLD) group (n=43). Liver damage was defined as hepatomegaly or the presence of any elevated liver function test. And from September 2017 to September 2020, 24 patients diagnosed as AOSD at the disease onset were enrolled in our prospective study, including 7 male patients and 17 female, aged (42.37 ± 12.79) years (range, 27~66 years). Among them, 13 patients were combined with liver dysfunction, 11 cases were not. And 20 healthy donors [5 males and 15 females, aged (37.51 ± 10.30) years (range, 20~57 years)] without any disease records were recruited from Health Care Centers. All the patients for prospective study were not subjected to any medical treatment at the time of blood samples obtained. The Institutional Review Board of our hospital approved this study, signed and written informed consents were obtained form all patients according to the recommendations of the Declaration of Helsinki.

**Table 1:** Clinical characteristics of adult onset still’s disease (AOSD) and healthy control (HC) groups.

Characteristics	HC (n=19)	AOSD (n=72)			
		LD (n=29)	NLD (n=43)	*p*-value	Total
Age, years	44.21 ± 12.57	34.00 ± 2.47	41.91 ± 2.25	0.023	38.72 ± 14.63
Sex, female/male	10/9	18/11	33/10	0.179	51/21
Fever	–	29 (100)	40 (93.02)	0.146	69 (95.83)
Skin rash	–	26 (89.66)	28 (65.12)	0.018	54 (75.00)
Pleurisy	–	7 (24.14)	4 (9.30)	0.105	11 (15.28)
Pneumonia	–	3 (10.34)	4 (9.30)	0.146	7 (15.28)
Pericarditis	–	9 (31.03)	4 (9.30)	0.019	13 (18.06)
Liver dysfunction	–	29 (100)	0 (0)	–	29 (40.28)
Splenomegaly	–	15 (51.72)	8 (18.60)	0.003	23 (31.94)
Lymphadenopathy	–	13 (44.83)	18 (41.86)	0.803	31 (43.06)
leukocytisis ≥15,000mm^2^	–	9 (31.03)	11 (25.58)	0.789	20 (27.78)
Sore throat	–	21 (72.41)	22 (51.16)	0.071	43 (59.72)
Myalgia	–	17 (58.62)	10 (23.26)	0.003	27 (37.5)
Abdominal pain	–	0 (0)	2 (4.65)	0.238	2 (2.78)
Systemic score	–	6.28 ± 1.79	3.41 ± 1.81	<0.0001	4.57 ± 2.28

### Systematic Grading of Adult Onset Still’s Disease Patients

The clinical data and laboratory test results of the AOSD patients were collected and analyzed. The disease activity score (range, 0~12) for each patient was assessed according to the criteria described by Pouchot et al. (19). Each of the following symptoms would be scored as 1 point: fever, rash, pleurisy, pneumonia, pericarditis, liver enlargement or liver dysfunction, spleen enlargement, lymph node enlargement, WBC count ≥15 ×10^9^/L, sore throat, muscle pain, and abdominal pain. AOSD patients were then divided into LD and NLD groups, symptoms of LD and NLD patients were further compared.

### Flow Cytometric Analysis

In the retrospective part, the percentages and absolute counts of lymphocytes as well as CD4^+^ and CD8^+^ subpopulations of T cells in peripheral blood were tested with BD Multitest 4-color TBNK reagent (Catalog No. 340503), and a BD FACS Caliber Flow Cytometer (BD Biosciences, San Diego, CA, USA) was used for analysis. To evaluate the levels of RIPK1 and RIPK3 in peripheral blood lymphocytes of AOSD patients, blood samples were obtained from 24 AOSD patients and 20 healthy controls (HCs). First, 100 µl peripheral blood was lysed by erythrocyte lysate; the remaining cells were then washed with PBS and fixed with BD fixation/permeabilization solution (Catalog No. 554714) for 20 min. After fixation/permeabilization, cells were washed by BD perm/wash buffer. Then, intracellular RIPK1/3 was stained with rabbit IgG anti-human RIPK1/3 antibody (Abcam, catalog No. 178420 for RIPK1 and US Biological, catalog No. 041063 for RIPK3) or control rabbit IgG and incubated for 30 min. Cells were then washed twice with PBS, and stained with a secondary phycoerythrin (PE)-conjugated anti-rabbit IgG antibody (BD Bioscience) for 30 min avoid from light. After washing, cells were suspended with 500 μl PBS and then analyzed on a BD FACS Aria II Flow Cytometer; the data were analyzed using FlowJo software (TreeStar, Inc., San Carlos, CA, USA).

### Histological Analysis

Liver biopsy specimens were obtained from two AOSD patients with liver injury. The samples were immobilized by 4% neutral formaldehyde solution and embedded in paraffin blocks. Liver sections underwent H&E and immunohistochemistry staining. For immunohistochemistry staining, the sides were first incubated with primary antibodies against RIPK1 (Abcam, catalog No. ab72139), RIPK3 (Abcam, catalog No. ab62344) overnight at 4°C. DAB substrate kit (BD Biosciences, USA) was then used for immunohistochemical staining, according to its protocol. Liver sections were then imaged with a microscope (Leica, Germany). Liver biopsy specimen from an autoimmune hepatitis (AIH) patient was used as disease control. The positive results of immunohistochemical were brownish yellow without background staining.

### Statistical Analysis

The quantitative data was shown as mean ± SD, differences between the two groups were analyzed by Student’s *t* test or the Mann–Whitney *U* test as appropriate; differences among three groups were analyzed by one-way ANOVA test. Normal distribution test and Pearson’s correlation or Spearman’s correlation were performed correlation test as appropriate. SPSS19.0 statistical software (SPSS Inc.; Chicago, IL, USA), R (AT&T Bell Laboratories, USA) and GraphPad Prism statistical software 6 (CA, USA) were used for analysis. *p*<0.05 was considered as statistically significant.

## Results

### Clinical Characteristics of Adult Onset Still’s Disease Patients

In our retrospective study, women accounted for the majority of AOSD patients. More than half of AOSD patients were complicated with fever, skin rash, and sore throat. LD patients were younger in age and showed higher disease activity than NLD patients (*p*=0.023 and *p*<0.0001, respectively). What’s more, LD patients were more easily accompanied with skin rash, pericarditis, splenomegaly, and myalgia (all *p*<0.05, **Table 1**).

### Adaptive Immune Disorders in Adult Onset Still’s Disease Patients

**Figure 1:**
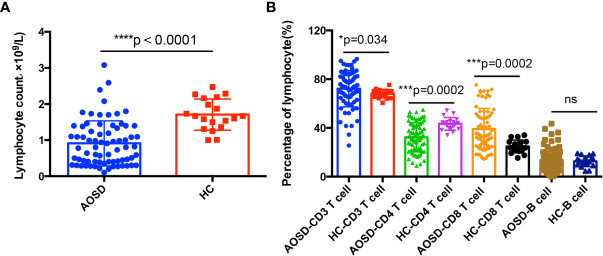
Adaptive immune disorders in adult onset still’s disease (AOSD) patients. Total lymphocyte counts **(A)** in AOSD patients are significantly lower than that of healthy controls (HCs). Lymphocytes subsets **(B)** distributions in AOSD and HCs, CD3^+^, and CD8^+^ T cells are significantly higher in AOSD group, while CD4^+^ T cells is lower in AOSD patients than that of HCs.

**Figure 2:**
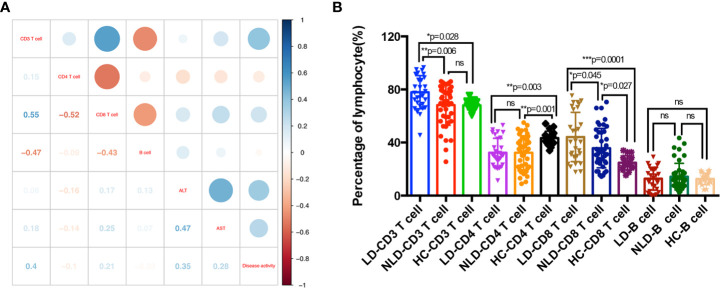
Adaptive immune disorders in adult onset still’s disease (AOSD) patients with LD and NLD. Correlations between percentage of lymphocytes and disease activity and liver function tests; numbers in the square were correlation coefficient **(A)**. Percentages of lymphocytes subsets in liver damage (LD), non-liver damage (NLD), and healthy control (HC) groups **(B)**. AST, aspartate aminotransferase; ALT, alanine aminotransferase.

As shown in **Figure 1**, AOSD patients showed abnormal lymphocytes distributions when compared with HCs. The total lymphocyte counts and percentage of CD4^+^ T cell decreased significantly (*p*<0.0001 and *p*=0.0002, respectively), whereas percentage of CD3^+^ and CD8^+^ T cells significantly increased (*p*=0.034 and *p*=0.0002, respectively) and B cells showed no statistical significance. The correlation between lymphocyte subsets proportions and disease activity and liver function tests were further analyzed. The percentage of CD3^+^ T cells was positively correlated with disease activity score (r=0.4, *p*=0.002). The CD8^+^ T cells proportion was positively correlated with aspartate aminotransferase (AST) (r=0.25, *p*=0.041) (**Figure 2A**).

**Figure 2B** showed the distributions of lymphocyte subsets in LD, NLD, and HC groups. The percentage of CD3^+^ T cells (*p*=0.0002), CD4^+^ T cells (*p*=0.0009), and CD8^+^ T cells (*p*=0.0002) showed statistical significance among the three groups. CD3^+^ T cells in LD group increased significantly than that of NLD and HC groups, whereas NLD showed similar CD3^+^ T cells proportion with HC (LD *vs.* NLD *vs.* HC, 77.93 *vs.* 68.28 *vs.* 68.09%). LD and NLD showed lower CD4^+^ T cells than that of HC (LD *vs.* NLD *vs.* HC, 32.34 *vs.* 32.32 *vs.* 43.35%). Both LD and NLD exhibited higher CD8^+^ T cells proportion than that of HC (LD *vs.* NLD *vs.* HC, 44.19 *vs.* 35.77 *vs.* 24.8%). What’s more, B cells showed no differences in lymphocyte distribution among the three groups.

### Increased RIPK1/3 Expression in Peripheral Blood Lymphocytes of Adult Onset Still’s Disease Patients

**Figure 3:**
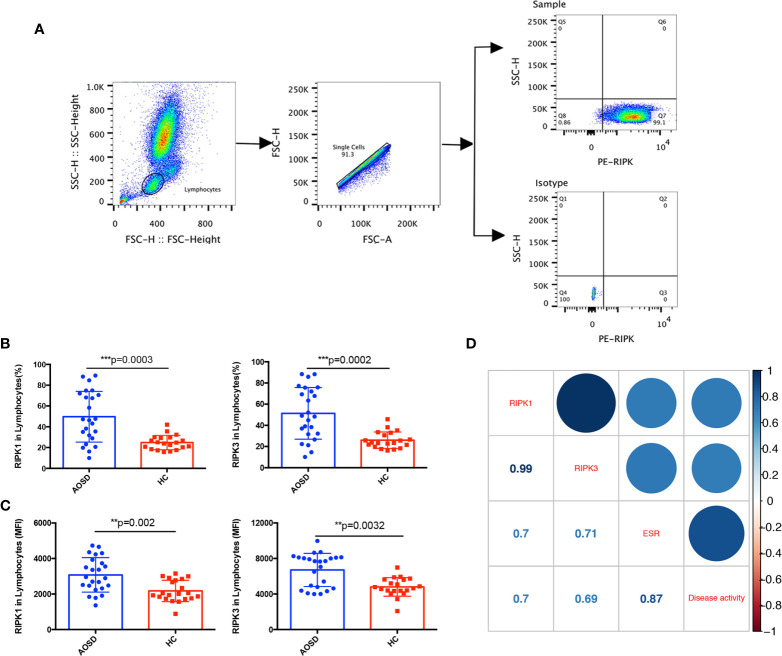
Increased RIPK1/3 expression in lymphocytes of adult onset still’s disease (AOSD) patients. The gating strategy of receptor interacting serine/threonine kinases (RIPKs) tested by flow cytometer **(A)**. Levels of RIPK1 and RIPK3 analyzed by lymphocyte (%) **(B)** and mean fluorescence intensity (MFI) **(C)** in lymphocytes were significantly higher in AOSD than that of healthy control (HC) group. Correlations between RIPK1/3 and erythrocyte sedimentation rate (ESR) and disease activity score of AOSD patients; numbers in the square were correlation coefficients **(D)**.

RIPK1/3 is not only related to apoptosis, but also can regulate the inflammatory response to auto-inflammatory diseases (11). As disorder of lymphocytes distributions was detected in AOSD patients of our retrospective study, we would like to explore whether abnormal RIPK1/3 expression was involved in the lymphocytes of AOSD patients. The gating strategy of flow cytometric analysis for RIPKs was depicted in **Figure 3A**. We first analyzed the correlation between RIPKs and lymphocyte counts. Both RIPK1 and RIPK3 showed negatively correlation with lymphocyte counts (r=−0.45, *p*=0.027 and r=−0.43, *p*=0.032 respectively). Differences of RIPKs between AOSD and HC were further compared, as shown in **Figure 3B**, RIPK1 (AOSD *vs.* HC, 49.68 *vs.* 24.77%) and RIPK3 (AOSD *vs.* HC, 51.3 *vs.* 25.97%) in lymphocytes of AOSD patients were significantly higher than that of HC group (*p*=0.0003 and *p*=0.0002, respectively). Differences of mean fluorescence level (MFI) of RIPK1/3 in AOSD and HC (**Figure 3C**) were also compared, and the results were consistent with the frequency in lymphocytes (%).

To further evaluate whether RIPK1/3 was associated with disease activity, the correlations among RIPKs, erythrocyte sedimentation rate (ESR), and disease activity score were analyzed. **Figure 3D** showed that RIPK1 and RIPK3 levels were positively correlated with ESR (r=0.7, *p*=0.0001 and r=0.71, *p*=0.0001 respectively) and disease activity score (r=0.7, *p*=0.0001 and r=0.69, *p*=0.0002 respectively).

### Receptor Interacting Serine/Threonine Kinases Are Involved in Liver Damage of Adult Onset Still’s Disease

**Figure 4:**
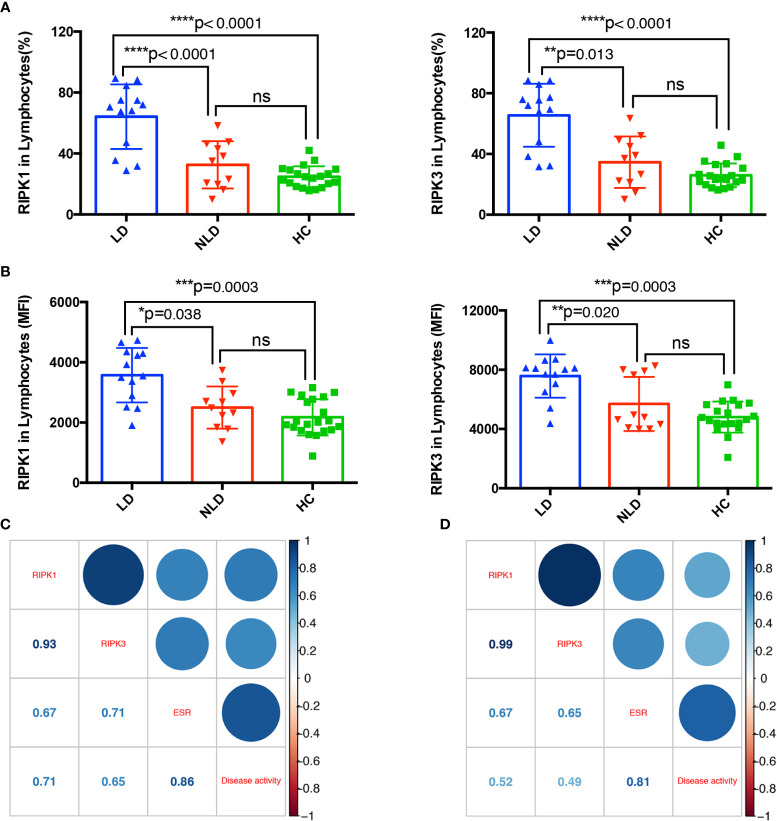
RIPK1/3 are involved in liver damage of adult onset still’s disease (AOSD). Levels of RIPK1/3 analyzed by lymphocytes (%) **(A)** and mean fluorescence intensity (MFI) **(B)** in liver damage (LD), non-liver damage (NLD), and healthy control (HC) groups. Correlation between receptor interacting serine/threonine kinases (RIPKs) and erythrocyte sedimentation rate (ESR) and disease activity score in LD **(C)** and NLD **(D)** groups. Numbers in the square were correlation coefficients.

In the prospective study, the 24 cases of AOSD patients were divided into LD group (13 cases) and NLD group (11 cases), and the frequencies of RIPK1/3 and MFI of RIPK1/3 in lymphocytes of LD, NLD, and HC were compared. The percentage of RIPK1 (LD *vs.* NLD *vs.* HC, 64.15 *vs.* 32.58 *vs.* 24.77%, *p* < 0.0001) and RIPK3 (LD *vs.* NLD *vs.* HC, 65.47 *vs.* 34.56 *vs.* 25.97%, *p* < 0.0001) showed significantly difference among the three groups. RIPK1 and RIPK3 in LD group were significantly higher than that of NLD or HC group, whereas no differences were detected between NLD and HC groups (**Figure 4A**). MFI of RIPK1/3 in the three groups showed consistent results (**Figure 4B**).

The correlations between RIPKs, ESR and disease activity in LD and NLD were then analyzed. In **Figures 4C, D**, RIPK1 and RIPK3 showed positive relationship with ESR and disease activity in both LD and NLD group

**Figure 5:**
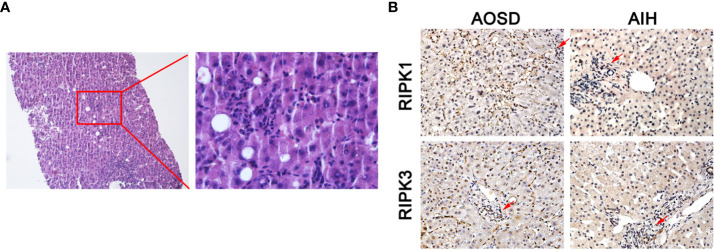
RIPK1/3 expressions in damaged liver biopsy of adult onset still’s disease (AOSD) patient. Considerable interstitial lymphocytes could be seen in the H&E staining of liver specimen from one AOSD patient **(A)**. Immunohistochemical analysis of liver biopsy specimens from AOSD and autoimmune hepatitis (AIH) patients. Interstitial lymphocytes infiltration (arrow) existed in both patients, but RIPK1/3 was mainly expressed in the cytoplasm of infiltrating lymphocytes and around the lymphocytes in liver of AOSD patient **(B)**.

The H&E staining of liver biopsy specimen from two AOSD patients showed considerable interstitial lymphocytic infiltrates (**Figure 5A**). Immunohistochemical staining indicated that interstitial lymphocytes infiltration (arrow top) existed in both AOSD patient with liver damage and autoimmune hepatitis (AIH) patient. RIPK1/3 was obviously expressed in and around lymphocytes of the liver samples from AOSD patients. But RIPKs were mainly detected in the cytoplasm of hepatocytes rather than lymphocytes in the AIH liver specimen (**Figure 5B**).

### Receptor Interacting Serine/Threonine Kinases as Biomarkers for Adult Onset Still’s Disease and Liver Damage Diagnosis

**Figure 6:**
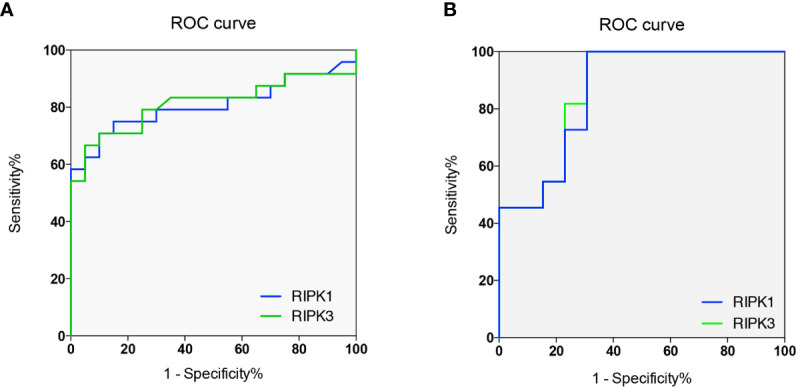
RIPKs as biomarkers for adult onset still’s disease (AOSD) and liver damage (LD) diagnosis. Receiver operating characteristic (ROC) curves of RIPK1 and RIPK3 to diagnose AOSD and LD. Areas under the curves (AUCs) were 0.671 and 0.813 respectively **(A)**. ROC curves of RIPK1/3 to predict liver damage of AOSD patients. AUCs were 0.867 and 0.860 respectively **(B)**.

To investigate whether RIPKs could be the potential markers for AOSD and LD diagnosis, ROC curve analysis was carried out; the cutoff values of RIPK1 and RIPK3 in peripheral blood lymphocytes (%) to best distinguish AOSD patients from HCs were determined (**Figure 6A**). For RIPK1, the area under curve (AUC) was 0.671 (95% confidence interval =0.671 to 0.943). Taking 33.7% as cutoff value, the sensitivity and specificity to identify an AOSD patients were 70.8 and 90%, respectively. And for RIPK3, the cutoff value was 38.3%, with sensitivity of 66.7%, specificity of 95%, and AUC was 0.813 (95% confidence interval =0.678 to 0.948).

To distinguish LD form NLD, ROC curve indicated that AUC for RIPK1 and RIPK3 were 0.867 (95% confidence interval =0.723 to 1.01) and 0.860 (95% confidence interval =0.712 to 1.01), respectively. For RIPK1, cutoff value were 69.2% with sensitivity of 100%, specificity of 69.2%; Taking 65.6% as cutoff value for RIPK3, the sensitivity and specificity were 100 and 69.2% for diagnosing LD in AOSD patients (**Figure 6B**). These results indicate that RIPKs can be promising biomarkers for LD diagnosis in AOSD.

## Discussion

Today, the etiology of AOSD is still unclear, and previous study showed that systemic inflammatory response played an important role in the pathogenesis of AOSD (3). Koeller et al. (20). indicated disorders in the number and function of immune cells in peripheral blood of AOSD patients including enlargement of lymph nodes and abnormal activation of T cells. A study from Jeon et al. *(* 21). showed the most common pathological types of lymph node from AOSD patients were paracortical hyperplasia with prominent vascular proliferation, scattered large B/T immunoblasts, and infiltration by reactive lymphocytes and inflammatory cells. These major pathology types along with our study suggested that the adaptive immune response mediated by T lymphocytes might play an important role in the pathogenesis of AOSD.

In recent years, more and more attention has been paid to the molecular mechanism and pathophysiology effects of programmed cell death. From routine clinical work, we found there was an abnormal T cell distribution in AOSD patients, which drive us to think that abnormal lymphocyte death might be involved in the process of the disease. RIPK1 and RIPK3 are the key proteins regulating cell death and inflammation, and their absence can completely block the occurrence of programmed cell death and participate in the regulation of various inflammatory signals (22). Therefore, RIPKs, as key molecules that regulate cell function, may play a role in the development of AOSD. Flow cytometric analysis revealed that the levels of RIPKs in peripheral blood lymphocytes of AOSD patients were negatively correlated with lymphocytes counts, and were positively correlated with ESR and disease activity score, which means that the higher RIPKs level were, the more serious the disease condition would be. RIPKs may participate in the development of AOSD inflammatory response by indirectly regulating the programmed necrosis of lymphocytes, and they may also activate a series of inflammatory reactions that independent from NF-κB signaling pathway (7).

Liver involvement is quite common in AOSD, LD patients of AOSD tended to be younger and to have higher disease activity score than NLD patients, which might be explained by younger patients having stronger immune responses. LD patients showed higher CD3^+^ and CD8^+^ T cells than NLD and HC groups, and NLD patients showed similar CD3^+^ T cells proportions to HCs. As RIPKs participate in many experimental liver disease models (9), we further explored RIPKs levels in peripheral blood lymphocytes and liver biopsy specimens in the prospective study. RIPK1/3 was significantly higher in AOSD blood sample than that of HCs. LD patients showed higher RIPKs expression than that of NLD and HCs, whereas NLD showed similar RIPKs expression with HCs. What’s more, RIPKs are positively correlated with ESR and disease activity score in both LD and NLD groups. Also, RIPKs were mainly detected in and around infiltrating lymphocytes in the liver of AOSD with LD, which was different from that of AIH. These results indicated that RIPKs might play a role in liver damage of AOSD. Cell death is an important driver of liver disease, while RIPKs were initially described as mediators of cell death and survival, emerging evidence indicates a necroptosis-independent role for the RIPKs in inflammation (9). Inhibition of RIPKs can decrease inflammatory response necroptotic cell death *in vitro* and *in vivo (* 23). Oral RIPK1 inhibitor has been reported to be useful in inflammatory diseases, including psoriasis, inflammatory bowel disease, and rheumatoid arthritis (24, 25). As RIPKs mainly expressed in lymphocyte in dysfunction liver of AOSD, the mechanism of liver damage mediated by RIPKs might be different from other kinds of liver dysfunction. Further studies are needed to clarify the pathogenic mechanism.

To date, there is no gold standard for AOSD diagnosis, though Yamaguchi’s criteria have widely been applied in clinics worldwide. And a few criteria and biomarkers such as serum ferritin, CXCL10, MIF, and so on have been proposed as biomarkers of AOSD diagnosis (4, 26). As mentioned above, RIPK1/3 level was significantly increased in AOSD, they might be the potential biomarkers for the diagnosis of AOSD, and ROC curve analysis showed favorable diagnostic value with AUC 0.671, 0.813 for RIPK1 and RIPK3 respectively. RIPK1/3 expression is significantly different in LD and NLD groups, so we further explored whether RIPKs can be used for liver damage diagnosis. From ROC curve analysis, the AUC for RIPK1 and RIPK3 to predict liver damage were 0.867 and 0.860 respectively. As RIPKs were correlated with ESR and disease activity score, they can be applied to monitor disease activity to a certain extent.

There are some limitations of this study. Firstly, the sample size in prospective study was quite small, which is mostly due to the low incidence of AOSD; more patients should be enrolled to make the cutoff values of RIPKs more precisely for diagnosis and evaluation of the disease. Secondly, there are no AOSD animal models, so animal experiments could not be conducted; and cellular experiments with RIPKs inhibitor will be performed in the future for mechanism research. Thirdly, as AOSD liver sample is very difficult to obtain, there were only two cases in our study; more cases should be enrolled to confirm the results.

In summarize, RIPK1 and RIPK3 levels in lymphocytes are significantly higher in AOSD patients, and are closely related to the disease activity and liver damage. RIPKs have the potential to become new biomarkers for diagnosis of AOSD and liver damage.

## Data Availability Statement

The original contributions presented in the study are included in the article/supplementary material. Further inquiries can be directed to the corresponding author.

## Ethics Statement

The studies involving human participants were reviewed and approved by Medical Ethical Committee of Renji Hospital, School of Medicine, Shanghai Jiao Tong University. The patients/participants provided their written informed consent to participate in this study.

## Author Contributions

LL designed the study revised the manuscript. XL and RG performed the experiments and drafted the manuscript. XM and JF collected and analyzed clinical data. All authors contributed to the article and approved the submitted version.

## Funding

This work was supported by the National Key Research and Development Program of China (2017YFC0909002), the National Natural Science Foundation of China (Grant No: 81373209 and 82001707), and Shanghai Sailing Program (20YF1425700).

## Conflict of Interest

The authors declare that the research was conducted in the absence of any commercial or financial relationships that could be construed as a potential conflict of interest.
